# Citrullinated Histone H3, a Marker for Neutrophil Extracellular Traps, Is Associated with Poor Prognosis in Cutaneous Squamous Cell Carcinoma Developing in Patients with Recessive Dystrophic Epidermolysis Bullosa

**DOI:** 10.3390/cancers16132476

**Published:** 2024-07-06

**Authors:** Hélène Ragot, Sonia Gaucher, Mathilde Bonnet des Claustres, Justine Basset, Rose Boudan, Maxime Battistella, Emmanuelle Bourrat, Alain Hovnanian, Matthias Titeux

**Affiliations:** 1 Laboratory of Genetic Skin Diseases, Imagine Institute, Université Paris Cité, INSERM UMR 1163, 75015 Paris, France; helene.ragot@inserm.fr (H.R.); sonia.gaucher@aphp.fr (S.G.); mathilde.bonnet-des-claustres@inserm.fr (M.B.d.C.); justine.basset@inserm.fr (J.B.); 2 Reference Center for Genodermatoses (“Maladies Génétiques à Expression Cutanée”, MAGEC), Saint-Louis Hospital (Assistance Publique—Hôpitaux de Paris), 75010 Paris, Franceemmanuelle.bourrat@aphp.fr (E.B.); 3 Department of Pathology, Saint-Louis Hospital (Assistance Publique—Hôpitaux de Paris), Université Paris Cité, 75010 Paris, France; maxime.battistella@aphp.fr; 4 Department of Genomic Medicine of Rare Diseases, Necker Hospital for Sick Children (Assistance Publique—Hôpitaux de Paris), Université Paris Cité, 75015 Paris, France

**Keywords:** recessive dystrophic epidermolysis bullosa, cutaneous squamous cell carcinoma, citrullinated histone H3

## Abstract

**Simple Summary:**

Recessive dystrophic epidermolysis bullosa (RDEB) is a rare and severe hereditary skin disease characterized by skin and mucosa fragility. RDEB patients are predisposed to the development of life-threatening cutaneous squamous cell carcinoma (SCC). In this study, we investigated the immune profiles of SCCs occurring in a cohort of RDEB patients and compared them with clinical, histopathological and prognostic features. We describe two distinct clinical outcomes in RDEB patients with cutaneous SCCs. A majority of RDEB patients had local and not rapidly life-threatening SCC, while others developed early aggressive and metastatic SCCs. The high-risk primary RDEB-SCC was associated with a tumor microenvironment displaying a higher neutrophil-to-lymphocyte infiltration ratio. Increased levels of citrullinated histone H3, a marker of neutrophil extracellular traps (NET), were detected in tumoral skin and in the serum of RDEB patients with high-risk primary SCC.

**Abstract:**

Recessive dystrophic epidermolysis bullosa (RDEB) is a rare severe hereditary skin disease characterized by skin and mucosa fragility, resulting in blister formation. The most severe complication in RDEB patients is the development of cutaneous squamous cell carcinoma (SCC), leading to premature death. There is a great deal of evidence suggesting a permissive tumor microenvironment (TME) as a driver of SCC development in RDEB patients. In a cohort of RDEB patients, we characterized the immune profiles of RDEB-SCCs and compared them with clinical, histopathological, and prognostic features. RDEB-SCCs were subdivided into four groups based on their occurrence (first onset or recurrences) and grading according to clinical, histopathological parameters of aggressiveness. Thirty-eight SCCs from 20 RDEB patients were analyzed. Five RDEB patients experienced an unfavorable course after the diagnosis of the first SCC, with early recurrence or metastasis, whereas 15 patients developed multiple SCCs without metastasis. High-risk primary RDEB-SCCs showed a higher neutrophil-to-lymphocyte ratio in the tumor microenvironment and an increased proportion of neutrophil extracellular traps (NETs). Additionally, citrullinated histone H3, a marker of NETs, was increased in the serum of RDEB patients with high-risk primary SCC, suggesting that this modified form of histone H3 may serve as a potential blood marker of unfavorable prognosis in RDEB-SCCs.

## 1. Introduction

Recessive dystrophic epidermolysis bullosa (RDEB) is an orphan genetic disease caused by mutations in *COL7A1* [1], leading to skin and mucosal blisters and erosions upon minor trauma. Skin fragility results in chronic inflammatory wounds or dystrophic scars in RDEB patients, which promote the development of cutaneous squamous cell carcinomas (SCCs). SCCs represent the main cause of death in RDEB patients due to their recurrent, sometimes multifocal, and early metastasizing characteristics [2,3]. The frequency, the number, and the size of SCCs seem to correlate with disease severity, with a higher prevalence for severe RDEB patients [2]. Although most RDEB patients develop early aggressive and metastatic lesions leading to rapid death, some patients developing not life-threatening focal SCCs have been reported [3,4,5]. To date, there is no marker predicting these two clinical developments.

The pathogenesis of SCCs in RDEB patients is not completely elucidated. Previous studies have suggested the implication of a permissive microenvironment in chronic wounds or lesional skin as a driving mechanism in SCC development [6,7]. Cutaneous blisters in RDEB patients have revealed high levels of pro-inflammatory cytokines and chemokines, as well as infiltrated cells, including T cells and myeloid cells [8]. Notably, plasmatic IL-1β levels have been shown to be increased in RDEB patients, which may promote immune suppression and facilitate tumor progression [9]. In contrast, investigations of the tumor microenvironment [10] of RDEB-SCCs have shown reduced infiltration of CD3+, CD4+, and CD68+ immune cells compared with sporadic SCCs [11,12,13], indicating an immunosuppressive TME in RDEB-SCCs.

It is now well established that cell–TME interactions play a key role in cancer development and metastasis [14,15]. Neutrophils, in particular, have emerged as important components of the TME. Indeed, they represent the most abundant type of innate immune cells in humans, acting as the first line of defense against pathogens by phagocytosis, degranulation, and/or releasing reactive oxygen species [16]. Neutrophil infiltration in the TME has been associated with tumor progression and metastasis in many cancer types by promoting tumor cell growth and immunosuppression [17,18]. Upon activation, neutrophils can release decondensed nuclear DNA–histone complexes associated with proteases and inflammatory mediators, known as neutrophil extracellular traps (NETs) [19]. NETs have been shown to contribute to cancer cell progression [20,21] and metastasis spread in mouse models [22,23,24] and patients [25,26]. NETs may promote metastasis by modulating epithelial–mesenchymal transition in tumor cells, regulating pre-metastatic niches, or leading to the release of circulating tumor cells [22,27,28]. NETs have been detected in primary tumors, metastatic tissues, and the patient circulatory system [20]. Studies have shown that infiltrated NETs in the TME correlate with a higher detection of NETs in the peripheral blood [26]. Finally, citrullinated histone H3, a marker of NETs [29], was found to be elevated in the blood of patients with advanced cancer and was associated with a poor prognosis [30,31].

This study aimed to investigate the clinical features in 20 RDEB patients included between 2015 and 2022 in a single French EB reference center. The immune profile in 38 SCCs was investigated and compared with clinical, histopathological and prognostic features to identify immune cell populations that could be associated with a poor prognosis.

## 2. Materials and Methods

### 2.1. Study Design and Ethics

The SIMOCEB study was approved by the Paris Nord Ethics Review Committee for Biomedical Research Projects (CEERB: n°2020–010/ClinicalTrials.gov: NCT04285294). Patients were followed in the MAGEC reference center (Centre de Référence des Maladies Rares de la Peau et des Muqueuses d’Origine Génétique Nord) at Saint-Louis Hospital. The study included a biological collection gathered retrospectively between March 2015 and December 2019, and a biological collection from patients included prospectively from January 2020 to December 2022 after written informed consents were obtained. The inclusion criteria were as follows: (1) age older than 18 years old, (2) confirmation of RDEB subtype, and (3) one or several SCCs surgically treated. The exclusion criteria were the following: (1) under protection by law and (2) absence of health insurance coverage. One RDEB-Sev patient was not included due to a non-cutaneous SCC localization (tongue) associated with tobacco smoking.

The confirmation of RDEB subtypes—severe RDEB (RDEB-Sev), intermediate RDEB (RDEB-Int), and inversed RDEB (RDEB-Inv)—relied on clinical findings according to the clinical practice guidelines [32], and was supported by type VII collagen expression by immunofluorescence and *COL7A1* mutation analysis performed by Next-Generation Sequencing or Sanger Sequencing.

The diagnosis of SCCs was established by the histopathological analysis of biopsies from suspicious skin lesions before surgery.

**Figure 1:**
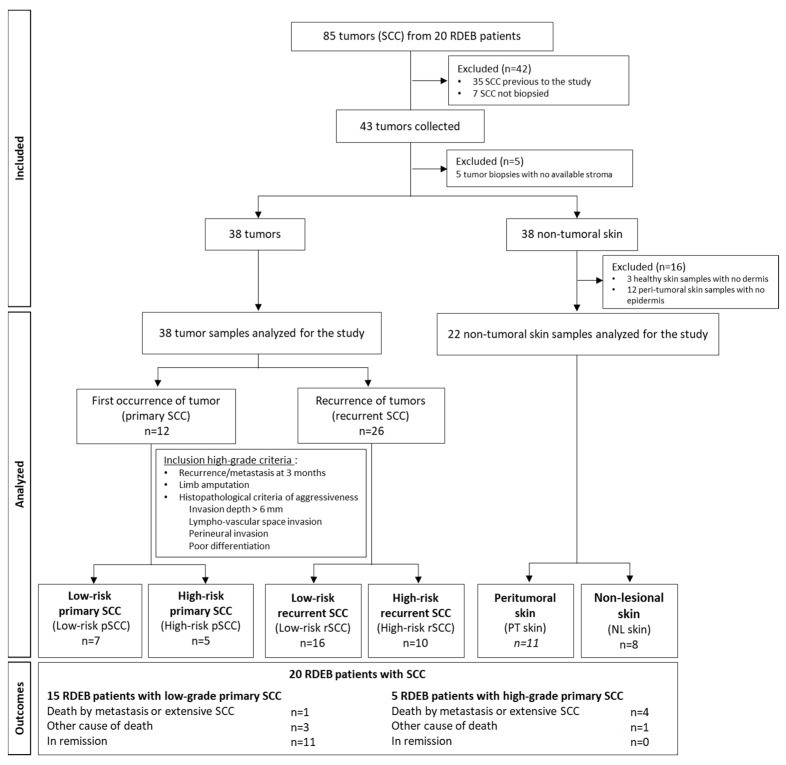
Flowchart showing the strategy and the grading criteria used to classify and investigate recessive dystrophic epidermolysis bullosa-cutaneous squamous cell carcinoma (RDEB-SCC) in the SIMOCEB study.

Tumor samples were obtained by biopsy prior to any treatment from 43 RDEB-SCCs. At the time of the surgery, 3 different types of samples were collected from surgical resections and residues using punch biopsies, when possible, from RDEB patients: the tumor, the lesional peri-tumoral skin, and the non-lesional skin for immunohistological and/or molecular analyses. Depending on the size of the tumor and surgical excision, not all biological samples could be collected from the 3 locations for the different experiments. As a result, the use of biopsies was prioritized based on the type of analysis required. A flow chart of the analyzed samples is described in Figure 1.

The morphological parameters of the 43 SCCs were defined by pathologists. Five SCCs were discarded due to an unsatisfactory stroma quality. The biological material available for the study is listed in Supplementary Table S1.

The diagnostic stage was based on the American Joint Committee on Cancer Staging Manual, 8th Edition classification system [33,34] to determine the TNM classification. High-risk RDEB-SCC features were validated by a dermatologist, pathologist, and surgeon according to the following criteria: local recurrence or metastasis within 3 months, massive local extension with recommendation of limb amputation, and histopathological criteria of aggressiveness (invasion depth > 6 mm, lympho-vascular space invasion, perineural invasion, poor differentiation). Four SCC groups were analyzed: low-risk primary SCCs (low-risk-pSCCs), high-risk primary SCCs (high-risk-pSCCs), low-risk recurrent SCCs (low-risk-rSCCs), and high-risk recurrent SCCs (high-risk-rSCCs). The proportion of aggressiveness parameters in high-risk SCC groups is listed in Supplementary Table S2.

### 2.2. Histology and Immunolabeling

Tissue samples were fixed in 4% formaldehyde in PBS pH 7.4 for 24 h, then embedded in paraffin. Hematoxylin and eosin (HE) staining was performed on 5 µm paraffin-embedded sections using standard histological techniques. Representative images were taken using a slide scanner system, Nanozoomer 2.0 HT (Hamamatsu), at the SFR Necker’s histology platform.

Immunostaining analyses were performed on 5 m paraffin sections of samples on the entire width of the punch biopsy, including 32 of the 38 analyzed RDEB-SCCs (n = 6/7 for low-risk primary SCCs, n = 5/5 for high-risk primary SCCs, n = 13/16 for low-risk recurrent SCCs, and n = 8/10 for high-risk recurrent SCCs). Sections were deparaffinized, rehydrated, and incubated in sodium citrate buffer (pH 6) for 45 min at 95 °C for antigen retrieval. Then, sections underwent a block of non-specific staining via incubation with a BSA 5% buffer for 30 min at room temperature (RT). The primary antibodies listed in Supplementary Table S4 or the corresponding IgG isotype, as a negative control, was incubated at 4 °C overnight. The following day, the appropriate secondary antibodies were incubated for 45 min at RT. Nuclei were stained using DAPI (1 μg/mL). The specificity of the antibodies was validated by two investigators (H.R. and M.B.d.C). Positive cells were quantified at 200× magnification under a Axiovert 200 microscope (Carl Zeiss, Jena, Germany). Two non-consecutive sections per sample were analyzed for each staining. Five to eight fields were captured along the invasive margin of the tumors or the papillary dermis of a non-tumoral skin sample as additional fields were sometimes required to improve the representative mean cell density. Positive cells per field were quantified using Fiji software (ImageJ version 1.53c, Java 1.80_172, 64-bit, NIH, Bethesda, MD, USA). Representative images were taken using a Leica TCS SP8 SMD confocal microscope at 400× or 630× magnification.

### 2.3. Relative Quantification of Gene Expression by Real-Time PCR

Samples for molecular analyses were collected from 24 RDEB-SCCs, including n = 6 for low-risk primary SCC, n = 3 for high-risk primary SCC, n = 7 for low-risk recurrent SCC, and n = 8 for high-risk recurrent SCC, as well as n = 8 for non-lesional skin and n = 7 for peri-tumoral skin.

Total RNA was extracted from 5 mm skin biopsies using an RNeasy Fibrous Tissue Mini Kit (Qiagen, Redwood, CA, USA). Reverse transcription was carried out with 500 ng of total RNA using a Super Script IV Reverse transcription kit (Life Technologies, Carlsbad, CA, USA). cDNA was amplified using Mesa Green qPCR kits for SYBR Assay (Eurogentec, Liège, Belgium) and Applied Biosystems 7300 Real-Time PCR System (Life Technologies). The primers for each target gene are listed in Supplementary Table S5. Relative gene expression was normalized to a housekeeping gene (PGK) and calculated using the 2^−ΔΔCt^ method.

### 2.4. Citrullinated Histone H3 ELISA

Histone H3 citrullination was measured in the serum of RDEB patients or healthy controls using the EpiQuik circulating Histone H3 Citrullination ELISA kit, according to the manufacturer’s instructions (#P-3097, EpiGentek, NY, USA). RDEB blood samples were collected during routine medical follow-up with or without SCC occurrence (n = 11 and n = 13 respectively) and were centrifuged for 5 min at 3000× *g* to collect the serum. Briefly, samples (30 µL) and diluted standards were added to the plate coated with an anti-histone H3 (Citrulline R2+R8+R17) antibody and incubated for 60 min at 37 °C. After three washes, the detection antibody solution was added to each well for 60 min at RT. The plates were then washed four times and incubated with the detection substrate solution for 10 min at RT in the dark. Finally, a stop solution was added and the signal was measured on an automated analyzer, Tecan (Research Triangle Park, NC, USA). The optical density was measured at 450 nm. Histone H3 citrullination concentration in the serum was calculated based on the optical density value of the histone H3R2R8R17cit_3_ standard curve. Measurements were always above the detection threshold and fell within the linear range of the standard curve.

### 2.5. Statistical Analysis

Analyses were conducted using the GraphPad Prism v10 software (La Jolla, CA, USA). Values are given as means ± standard error of the mean. Each dot represents the mean of the quantifications for one sample. A Shapiro–Wilk normality test was used to assess whether the data followed the Gauss law. Depending on the normality of the dataset, statistical differences between the studied groups were assessed by Kruskal–Wallis or ANOVA tests, followed by Dunn’s or Tukey’s multiple comparison test. The Cox proportional hazard assumption was tested in clinicopathological parameters, and multivariate Cox regression was conducted to further visualize the influence of the parameters on the prognosis of the patients. Differences were considered significant for *p* < 0.05.

## 3. Results

### 3.1. Two Distinct Clinical Outcomes of RDEB Patients with SCCs

From March 2015 to December 2022, 20 RDEB patients were included, representing a total of 85 clinically reported cutaneous SCCs. The disease characteristics of the patients are described in Table 1 and Supplementary Table S1.

The RDEB subtypes included 16 RDEB-Sev (80%), 2 RDEB-Int (10%), and 2 RDEB-Inv (10%) subtypes. The median age of onset of the first SCC was 29 years (range: 18–48 years). Eighteen out the 20 RDEB patients developed at least one recurrent SCC with a median of 5.1 SCCs per patient (range: 1–17) and a median frequency of recurrence of 14 months (range: 1–85 months). The median survival after the first SCC occurrence was 4.6 years (range: 0.4–19.1 years) regardless of the RDEB subtype. The median survival was 2.3 years for RDEB patients with the RDEB-Sev subtype (range: 0.4–14.6 years) in line with a previous study [3]. None of the RDEB patients received preoperative treatments, including chemotherapy, radiotherapy, or immunotherapy.

High-risk primary SCC (high-risk pSCC) was observed in 5 RDEB-Sev patients. Four of these patients developed a high-risk recurrence, locally or by metastasis within 3 months, leading to death by extensive SCCs or metastasis of the patients. The fifth patient died from sepsis not related to his SCC. The median survival of RBEB patients with high-risk pSCC was 1.2 years (range: 1.1–2.2 years).

In contrast, 15 RDEB patients presented low-risk primary SCC (low-risk pSCC) without aggressive criteria (11/15 RDEB-Sev, 2/2 RDEB-Int, 2/2 RDEB-Inv). Four of these patients developed one high-risk recurrent SCC (high-risk rSCC), leading to death from metastasis in 1 patient 8 months after the high-risk rSCC diagnosis. Three RDEB patients died from complications unrelated to their SCC, including 1 patient by sepsis 5 months after his low-risk pSCC. To date, 11 patients are in remission. The median overall survival of the 15 patients with low-risk pSCC was significantly higher than that of the 5 patients with high-risk pSCC (9.3 years; range: 0.4–19.1 years) (*p* = 0.003 by Mann–Whitney test).

### 3.2. Clinical, Histopathological Features of RDEB-SCCs

Biological tissues were collected from 43 out of the 85 SCCs (Table 1). RDEB-SCCs were predominantly located on the extremities (31/43; 72%). Based on the histological reports mentioning the size of the tumor, 27 SCCs had a size greater than 2 cm (27/36; 67.5%), a factor of the T2 stage in the TNM classification [33]. An invasion deeper than 6 mm was measured in 7 of 39 SCCs (16.3%), whose extension into the dermis was mentioned. Good differentiation was observed in 28 SCCs (28/43; 65.1%), while 15 SCCs exhibited moderate to poor differentiation (34.9%). Most patients were initially diagnosed with stage II skin cancer (39/43; 90.7%). The prognostic power of clinicopathological parameters was evaluated with Cox proportional hazards regression analyses in association with prognosis of RDEB patients with a primary SCC (Supplementary Table S3). As expected, a poor differentiation state of SCCs correlated with severe prognosis in (multi)variate Cox regression.

**Figure 2:**
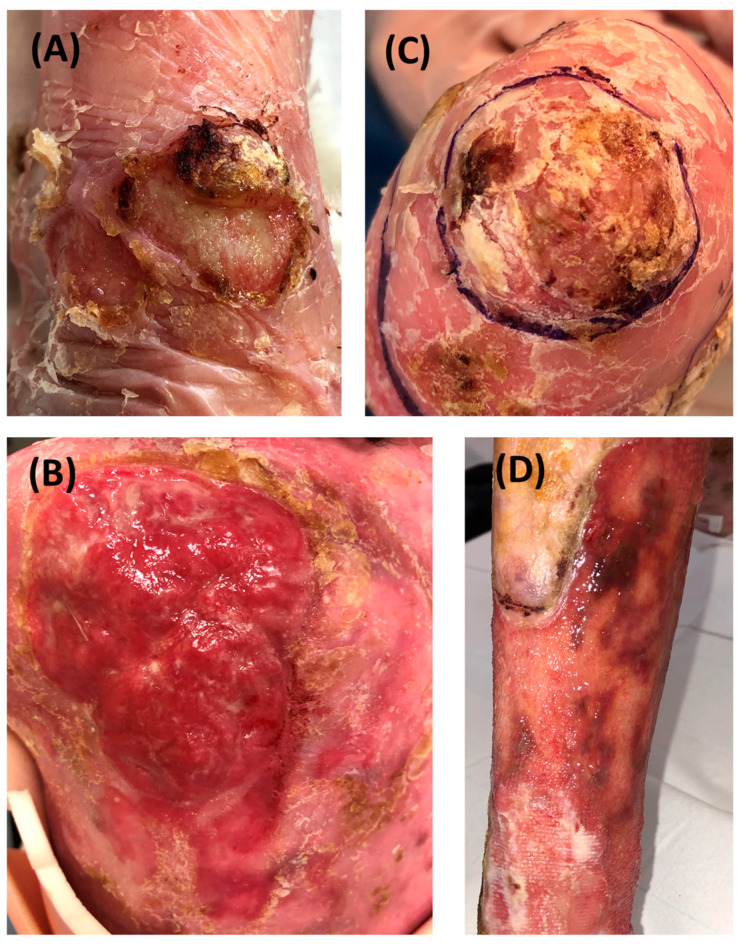
Representative clinical pictures of RDEB-SCCs in the different groups. (**A**) Low-risk primary SCC developed on the wrist. The tumor is circled. (**B**) low-risk recurrent SCC developed on the hand with hyperkeratosis. (**C**) Extensive high-risk primary SCC on the back. (**D**) Extensive high-risk recurrent SCC on the shin developed 2 months after the excision of a high-risk primary SCC.

RDEB-SCCs were divided into four groups according to their grading determined by clinical, histopathological parameters and recurrence (Figure 1 and Supplementary Table S2). Representative clinical pictures of RDEB-SCCs from each subgroup are depicted in Figure 2.

**Figure 3:**
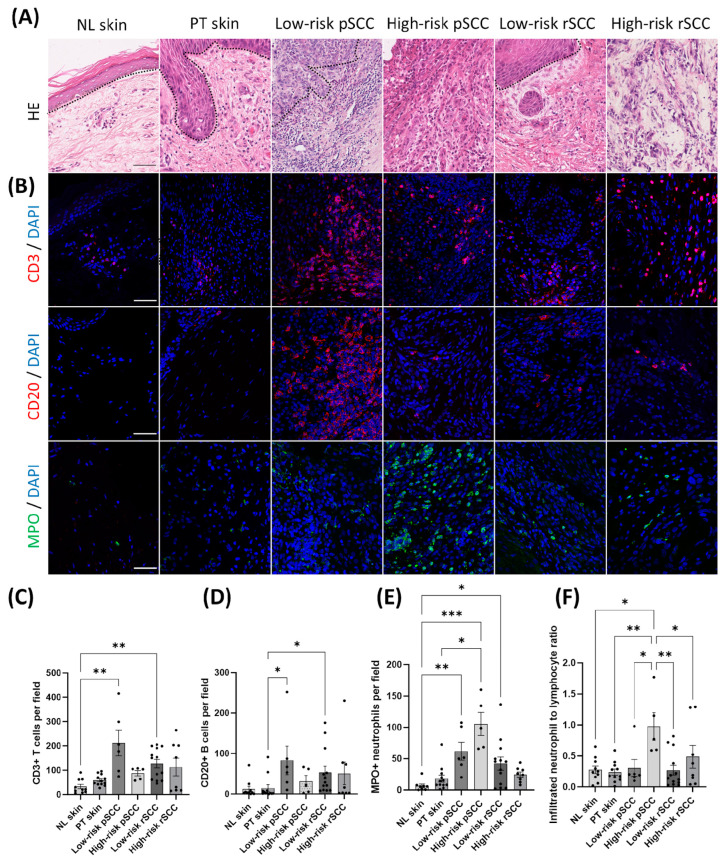
Histopathological features and detection of immune cells in RDEB-SCCs with different severity. (**A**) Representative hematoxylin–eosin staining (HE) in non-lesional skin (NL skin), peri-tumoral skin (PT skin), low-risk primary SCCs (low-risk pSCCs), high-risk primary SCCs (high-risk pSCCs), low-risk recurrent SCCs (Low-risk rSCCs), and high-risk recurrent SCCs (high-risk rSCCs) from RDEB patients. (**B**) Representative immunostaining of infiltrated CD3+ T cells, CD20+ B infiltrated lymphocyte cells, and myeloperoxidase (MPO)+ neutrophils in the different conditions. Scale bar = 50 µm. Quantification of the number of positive cells per field for CD3+ T cells (**C**), CD20+ B cells (**D**), MPO+ neutrophils (**E**), and number of positive cells for neutrophil-to-lymphocyte infiltrating cells (**F**) (n = 10 for NL skin, n = 12 for PT skin, n = 6 for low-risk pSCC, n = 5 for high-risk pSCC, n = 13 for low-risk rSCC, n = 8 for high-risk rSCC). * *p* < 0.05, ** *p* < 0.01, *** *p* < 0.001 Kruskal–Wallis test with Dunn’s or ANOVA with Tukey’s multiple comparison post hoc. Data are means ± SEM.

Representative HE stainings of the different groups are shown in Figure 3A. Most of the low-risk pSCCs (5/6) and all low-risk recurrent SCCs (low-risk rSCCs) (16/16) were well differentiated at the initial diagnosis. Conversely, 4 of the 5 high-risk pSCCs showed poor differentiation, while 1 high-risk pSCC was well differentiated. One RDEB patient developed 2 synchronous primary SCCs in the same location, one moderately differentiated low-risk pSCC and one poorly differentiated high-risk pSCC. The majority of high-risk rSCCs (7/10) originated from local recurrences or metastases of previously resected high-risk SCCs. One high-risk rSCC arose from a prior tumor not included in our cohort, for which histological parameters were not available. Two high-risk rSCCs were classified as new tumors. Three out of the 10 high-risk rSCCs were poorly differentiated, while 4 showed moderate differentiation and 3 were well differentiated. Ki67-positive nuclei were detected mainly in the outer layers of the tumor nests in low-risk pSCCs and low-risk rSCCs (Supplementary Figure S1A). The number of Ki67-positive tumor cells was significantly higher in both high-risk pSCCs and high-risk rSCCs compared with non-lesional skin (Supplementary Figure S1B). Finally, the transcriptional expression of epithelial differentiation markers, *CDH1*, *OCLN,* and *KRT1* [35,36,37], was decreased in high-risk pSCCs compared with non-lesional skin or peri-tumoral skin (Supplementary Figure S1C).

### 3.3. Infiltrating Neutrophil-to-Lymphocyte Ratio Is Increased in High-Risk Primary SCCs in RDEB Patients

The TME of SCCs, peri-tumoral skin, and non-lesional skin of RDEB patients with different clinical, histopathological features was investigated by assessing immune cell infiltration (Figure 3 and Supplementary Figure S2).

Infiltrating lymphocytes were exclusively detected in the invasive margin of the tumors (Figure 3A,B). Very few immune cells infiltrated the intra-tumoral region in the SCC groups. The density of immune cells was quantified in the invasive margin of the tumors or in the stromal region of non-tumor samples. The density of CD20+ B cells and CD3+ T cells was increased in low-risk pSCCs and low-risk rSCCs compared with peri-tumoral skin and non-lesional skin, respectively (Figure 3C,D). No significant difference was noted in the proportion of CD4+ helper T cells and CD8+ cytotoxic T cells relative to CD3+ T cells in the different groups (Supplementary Figure S2B,C).

Innate immune cells were also detected in the invasive margin of the tumors (Figure 3B and Supplementary Figure S3). Specifically, the number of infiltrated CD163+ macrophages was increased in low-risk rSCCs compared with peri-tumoral skin and non-lesional skin (Supplementary Figure S3B). The density of tryptase+ mast cells was not different among the different groups (Supplementary Figure S3C). Conversely, the density of myeloperoxidase (MPO)-positive neutrophils was significantly increased in low-risk pSCCs, high-risk pSCCs, and low-risk rSCCs compared with non-lesional skin (Figure 3E). Finally, the ratio of tumor-infiltrated neutrophils to lymphocytes (defined by CD20+ or CD3+ cells), which has previously been associated with poor prognosis in several cancers [38], was specifically increased in high-risk pSCCs (Figure 3F).

### 3.4. Increased Pro-Inflammatory Mediators and Neutrophil Extracellular Traps in the TME and the Serum of RBEB Patients with High-Risk Primary SCC

**Figure 4:**
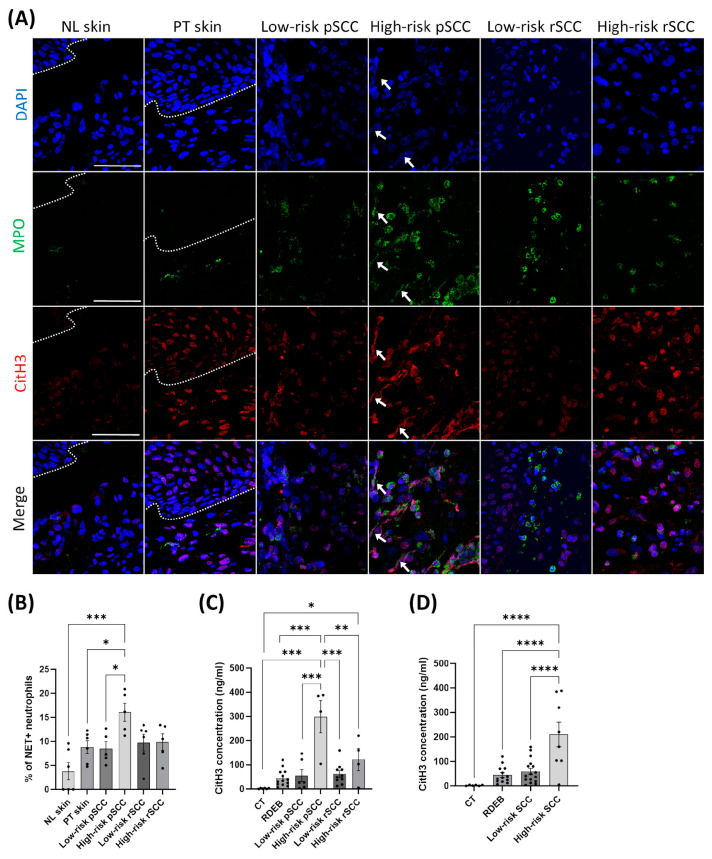
Detection of citrullinated histone H3, a marker for neutrophil extracellular traps, in the tumor microenvironment [10] and in the blood of RDEB patients with SCCs of different severities. (**A**) Representative immunostaining for myeloperoxidase (MPO) and citrullinated histone H3 (citrullination R2+R8+R17) (citH3) in the different conditions. White arrows indicate the presence of neutrophil extracellular traps positive for MPO, citH3 and DAPI (DNA) staining, scale bar = 50 µm. (**B**) Quantification of neutrophil extracellular trap formation measured by the proportion of NET-positive neutrophils to the total neutrophils in the different conditions (n = 5 to 6 per group). (**C**) Quantification of citH3 concentration in the blood of healthy controls (CT; n = 6), RDEB patients with no SCC (RDEB; n = 13), and RDEB patients with SCCs (n = 6 for low-risk pSCC, n = 4 for high-risk pSCC, n = 10 for low-risk rSCC, and n = 4 for high-risk rSCC). (**D**) Quantification of citH3 concentration in the blood of all RDEB patients by combining low-risk SCCs and high-risk SCCs. * *p* < 0.05, ** *p* < 0.01, *** *p* < 0.001, **** *p* < 0.0001 Kruskal–Wallis test with Dunn’s or ANOVA with Tukey’s multiple comparison post hoc. Data are means ± SEM.

Neutrophils have been shown to promote cancer dissemination through the formation of NETs in the TME [39]. To assess whether neutrophil infiltration in the TME of RDEB-SCCs was associated with NET formation, we performed immunostaining for MPO and citrullinated histone H3 (citH3), which are specific markers for neutrophils and NETosis [40], respectively, and with DAPI for DNA (Figure 4A). We found increased NET formation in high-risk pSCCs compared with low-risk pSCCs, peri-tumoral skin, and non-lesional skin, as measured by co-staining for MPO, citH3, and DAPI (Figure 4B).

Several studies have reported that NET formation can be mediated by various stimuli, including Interleukin-1β (IL-1β/*IL1B*) [41,42] and High-mobility group box 1 (HMGB1/*HMGB1*) [43], as well as by regulators of neutrophil recruitment including Interleukin-8 (IL-8/*CXCL8*) [41,44] and Granulocyte-macrophage colony-stimulating factor (GM-CSF/*CSF2* or G-CSF/*CSF3*) [45,46]. Quantitative RT-PCR analyses, performed on three high-risk pSCCs for which a sample was available, revealed higher levels of *CSF2* or *CSF3* transcripts (2/3) or both (1/3) (Supplementary Figure S4A,B). *IL1B* expression was also increased in high-risk pSCC and high-risk rSCC samples, with the expression of *CXCL8* and PADI4 being specifically increased in the high-risk pSCC group (Supplementary Figure S4C,D). Conversely, *HMGB1* expression was decreased in low-risk pSCCs and low-risk rSCCs compared with non-lesional skin (Supplementary Figure S4E).

Finally, we measured the concentration of citH3 in the serum of the RDEB patients with or without SCC to define whether circulating citH3 may be associated with cancer progression, as previously described [29]. The concentration of the NET marker citH3 was increased in the blood of RDEB patients with high-risk pSCC in comparison with RDEB patients with low-risk pSCC (Figure 4C). By combining patients with primary and recurrent SCCs, we found increased blood levels of citH3 in RDEB patients with high-risk SCC compared with patients with low-risk SCC (Figure 4D).

## 4. Discussion

Our study aimed at better characterizing the immune TME of SCCs in a cohort of 20 RDEB patients and comparing these results with clinical, histopathological features.

Consistent with the literature, our data support the notion that SCCs develop in RDEB patients from an early age with a median age 29 years at the initial diagnosis, and display a high risk of recurrence observed in 90% of RDEB patients [3,5]. We found a median survival of 4.6 years after the diagnosis of the first SCC, considering all RDEB subtypes and 2.3 years for the RDEB-Sev subtype. Our observations are in line with previous studies based on American, English, Australian, and Dutch EB registries (i.e., 2.4 years, 4 years, and 3.5 years, respectively) [2,5,47,48]. The severe RDEB subtype has previously been found to have the highest cumulative risk of death after the first SCC occurrence [3]. Previous studies have shown that most RDEB patients developed invasive, recurrent, or metastatic life-threatening SCC, rather than fewer patients having a prolonged phase of localized tumor growth [3,4,5]. These two distinct clinical outcomes were observed among patients with a similar clinical severity in the cohort we studied. However, RDEB patients with a more favorable disease course were the most numerous. Specifically, 15 out of 20 patients developed several recurrent SCCs with an overall median survival of 9.3 years. This relatively long period is in accordance with a previous study of the Spanish EB registry. Conversely, 5 RDEB-Sev patients developed several aggressive recurrences or metastases after their primary SCC occurrence, leading to a median survival of 1.2 years. Four of these RDEB patients with severe prognosis developed a poorly differentiated primary SCC, suggesting an early aggressive tumor. Therefore, new biomarkers need to be identified to predict a potential poor prognosis after the occurrence of the first SCC. Some potential tissue biomarkers have been associated with poor prognosis in RDEB-SCCs, including miR-10b or periostin [49,50]. A recent study reported miRNA signatures of RDEB-SCC-derived exosomes as potential circulating diagnostic biomarkers [51]. By contrast, no circulating prognostic biomarkers have been identified to predict the two distinct clinical outcomes of SCCs in RDEB patients.

To determine whether a specific immune TME could be associated with distinct clinical, histopathological features, we explored the immune cell infiltration in SCCs occurring in our RDEB cohort. The analyses highlighted increased neutrophil infiltrates compared with lymphocytes in the invasive margin of high-risk pSCCs. Neutrophils are the first cells to respond to inflammatory or infectious conditions. The accumulation of neutrophils in the TME has previously been reported as a factor of poor prognosis in different cancer types [52,53]. Likewise, an increased concentration of circulating neutrophils and an elevated infiltrating neutrophil-to-lymphocyte ratio have been considered as poor prognostic factors in cancers, including SCCs [52,54,55,56].

Neutrophil infiltration may result from a response to increased bacterial invasion in RDEB-SCCs. Consistent with this possibility, skin wounds of RDEB patients commonly show increased colonization and infections with different bacteria [57,58]. Furthermore, signatures of enhanced antibacterial immunity were identified in RDEB-SCCs, suggesting that the response to bacteria may be associated with an aggressive progression in RDEB-SCCs [59]. Previous studies have shown that infiltrated neutrophils in the TME could favor cancer cell growth and metastasis through the secretion of tumor growth-promoting factors [60,61] or the inhibition of the immune response [62]. In our study, we found no significant B and T cell infiltration in the TME of high-risk pSCCs and rSCCs. These observations are in line with recent studies that described a low immune cell infiltration in RDEB-SCCs in comparison with sporadic SCCs, with lower CD4+ and CD8+ cell infiltration [11,12,13].

Neutrophils may also participate in cancer progression with NET formation through systemic inflammation and thrombosis [21,22,23]. Increased blood levels of citH3, the marker of NETs, have been found to be a prognostic marker for endotoxic shock, suggesting that citH3 was a potential therapeutic target in this severe condition [63]. By contrast, several studies identified NETs and their components as direct drivers of epithelial–mesenchymal transition in various types of tumor cells, including human colon cancer cells [27] and breast cancer cells [24,39]. Furthermore, NETs can promote the spread and the metastasis of tumor cells by capturing tumor cells in the circulatory system [64], modulating pre-metastatic niches [28], or degrading the extracellular matrix around the tumor tissue by proteases [65]. The presence of NETs was previously described in the lesional RDEB skin and RDEB-SCCs [64]. Here, NETs tended to increase in the peri-tumoral skin but were significantly increased in high-risk pSCCs. These results are in agreement with those of another clinical study that identified that the presence of NETs in cancer tissues related to clinical stages and lymph node invasion [27]. Additionally, we found that NET formation in the TME of high-risk pSCCs was associated with increased blood levels of the NET marker citH3, which is consistent with observations in other types of cancer [30,66]. Therefore, our results support the potential association of increased levels of circulating biomarkers of NETs with adverse clinical outcomes, as already identified in different cohorts of patients with aggressive cancers [30,31,67]. In contrast, we did not find increased NET formation in the TME of high-risk rSCC. This difference could be explained by the fact that the majority of high-risk rSCCs (7/10) were local recurrences or metastases, all from previously resected high-risk SCCs. These data suggest that neutrophil infiltration may be dynamic with transient NET formation in the TME during the early stage of aggressive tumor progression. Additional mechanisms associated with aggressiveness may be involved in the reactivation of tumor cells in later stages. Nevertheless, RDEB patients with high-risk SCCs displayed elevated citH3 levels in their blood, compared with patients with low-risk SCCs or no SCCs. This result suggests that circulating citH3 could serve as a biomarker of poor prognosis.

Different mechanisms have been identified as drivers of NET formation, indirectly by the immune cells in the TME [68], or directly by the tumor cells by releasing various molecules such as IL-1, IL-6, IFN, TNF-α, C3a, HMGB1, or G-CSF [40,69,70]. To investigate the underlying molecular mechanisms involved in high-risk pSCCs, we measured transcript levels of pro-inflammatory markers related to NET activation in the tumors. The expression of a *HMGB1* transcript, a gene encoding a danger-associated molecular pattern, was not increased in RDEB-SCCs. This result is in agreement with published RNA-seq data from other investigators [71], but contrasts with the results of other studies showing increased levels of HMGB1 proteins in the blister fluid, lesional RDEB skin, and RDEB-SCCs [11,72]. Conversely, we found increased transcript levels of IL-1β, IL-8, and PAD4 associated with an enhanced expression of GM-CSF and/or G-CSF in high-risk pSCCs. IL-1β has been shown to drive NET formation through IL-8 or G-CSF production in inflammatory diseases including cancers [41,42,44], but also to directly target epithelial–mesenchymal transition [73]. The relationship between NETs in primary tumors and the poor prognosis in RDEB-SCCs was not investigated in this study. Additional investigations at the single-cell level are needed to identify the cell population responsible for signaling pathways underlying the tumor progression and aggressiveness in RDEB-SCCs, i.e., NET-associated signaling pathway, tumor growth, EMT, or cell dissemination. The main limitation of our study is the small sample size associated with high-risk pSCC in our cohort. This limitation was inherent in the orphan nature of the disease. Nevertheless, the present study, based on a nationwide recruitment for 7 years, represents one of the most important cohorts of this rare condition. The relevance of circulating citH3 as a prognostic biomarker for RDEB patients with SCCs should be confirmed by a prospective study of RDEB patients with primary SCCs. Therefore, a systemic dosage of citH3 in the blood of additional RDEB patients before SCC resection could be performed to validate the possible association between NET biomarker levels and the clinical outcome of the patients. Furthermore, the value of increased circulating citH3 as a reliable marker of local and/or systemic inflammation needs to be confirmed.

Due to the dominant role of NETs in an adaptive pro-tumor immune escape, the inhibition of NETs has been proposed as targets for cancer therapy [74]. A previous study in mice have shown that the pharmacological inhibition of PAD4 delayed tumor progression and could sensitize cancer cells to combined immunotherapy with anti-PD-1 and anti-CTLA-4 checkpoint inhibitors [75]. Reactive oxygen species oxygens play a crucial role in NET formation. Consequently, inhibiting metabolic mediators like NADPH oxidase via apocynin reduces NET formation in vitro [76]. Metformin, a first-line medication for diabetes known to inhibit the PKC–NADPH oxidase axis, has been shown to block NETosis in vitro [77]. Additionally, a recent study suggests that metformin may exert anti-neoplastic properties in aggressive RDEB-SCCs [78]. Therefore, it may be relevant to evaluate the potential of a local or topical application of molecules targeting neutrophil activation, such as metformin, as an adjuvant treatment of aggressive RDEB-SCCs resected with high systemic citH3 levels.

## 5. Conclusions

In conclusion, the present study describes two distinct clinical outcomes in RDEB patients with cutaneous SCCs. RDEB-SCCs with the poorest prognosis were associated with a higher neutrophil-to-lymphocyte infiltration ratio associated with an IL-1β-related signature. We showed increased levels of systemic citH3, a marker of NETs, in the serum of RDEB patients with the most aggressive SCCs, suggesting that citH3 could be a putative marker of unfavorable prognosis in RDEB-SCCs. In addition, albeit with limitations due to the sample size investigated, our study suggests that NETs are potential therapeutic targets to decrease the risk of local recurrence or metastasis in RDEB patients with SCC. Further studies with larger case series are therefore needed to validate the relevance of the biomarker citH3 in the prognosis and the management of RDEB patients with SCC.

## Figures and Tables

**Table 1:** Detailed clinical and histopathological features of RDEB patients and their SCCs studied.

RDEB Patients (n = 20) Sex (Male/Female)	6/14
**RDEB subtype**	
RDEB-Sev; n (%)	16 (80.0)
RDEB-Int; n (%)	2 (10.0)
RDEB-Inv; n (%)	2 (10.0)
**Follow-up**	
Median (range) age at first diagnosis in years	29 (18–48)
Median (range) number of SCCs by patient	5.1 (1–17)
Median (range) recurrence interval in months Median (range) survival after 1st SCCs in years Median (range) survival after 1st SCCs in RDEB-Sev in years Deaths by metastasis or extensive SCC; n (%) Other causes of death; n (%) In remission; n (%)	14 (1–85) 4.6 (0.4–19.1) 2.3 (0.4–14.6) 5 (25.0) 4 (20.0) 11 (55.0)
**SCC (n = 43)** **Location**	
Lower limb; n (%)	20 (46.5)
Upper limb; n (%)	11 (25.6)
Back; n (%)	9 (20.9)
Neck; n (%)	3 (6.9)
Size	
<2 cm; n (%)	6 (14.0)
≥2 cm < 4 cm; n (%)	16 (37.2)
≥4 cm; n (%)	14 (32.5)
NS	7 (16.3)
**Invasion depth**	
<3 mm; n (%)	16 (37.2)
≥3 mm < 6 mm; n (%)	16 (37.2)
≥6 mm; n (%)	7 (16.3)
NS	4 (9.3)
**Histopathological characteristics**	
Well differentiated; n (%)	28 (65.1)
Moderately differentiated; n (%)	9 (20.9)
Poorly differentiated; n (%)	6 (14.0)
**Lympho-vascular space invasion**	
Yes; n (%)	1 (2.3)
No; n (%)	42 (97.7)
**Perineural invasion**	
Yes; n (%)	2 (4.6)
No; n (%)	41 (95.3)
Clinical stage *	
I; n (%)	2 (4.6)
II; n (%)	39 (90.7)
III; n (%)	2 (4.6)
IV; n (%)	0 (0)

NS, not specified; SCC, cutaneous squamous cell carcinoma. * According to AJCC8 and Brigham and Women’s Hospital [33,34].

## Data Availability

Data are contained within the article and Supplementary Materials.

## Supplementary Materials

The following supporting information can be downloaded at https://www.mdpi.com/article/10.3390/cancers16132476/s1. Supplementary Figure S1: Detection of Ki67 and epithelial gene markers in RDEB-SCCs with different severities. Supplementary Figure S2: Detection of adaptive immune cells in RDEB-SCCs with different severities. Supplementary Figure S3: Detection of innate immune cells in RDEB-SCCs with different severities. Supplementary Figure S4: Gene expression of inflammatory mediators involved in neutrophil activation by quantitative RT-PCR. Supplementary Table S1: Supplementary RDEB patient and SCC tumor sample details. Supplementary Table S2: Aggressiveness parameters in high-risk SCC groups. Supplementary Table S3: Cox proportional hazards regression of clinical and histopathological variables in association with prognosis of RDEB patients with a primary SCC. Supplementary Table S4: Listing of antibodies. Supplementary Table S5: Listing of primers used for RT-quantitative PCR.
