# Supplementary material for: Citrullinated Histone H3, a Marker for Neutrophil Extracellular Traps, Is Associated with Poor Prognosis in Cutaneous Squamous Cell Carcinoma Developing in Patients with Recessive Dystrophic Epidermolysis Bullosa
Source: Cancers (Basel). 2024 Jul 6;16(13):2476. doi: 10.3390/cancers16132476 (PMC11240819; doi:10.3390/cancers16132476)
Supplement: Supplementary file 1 [file cancers-16-02476-s001.zip › cancers-3050482-supplementary.pdf]

Supplementary information

Supplementary tables

Supplementary Table S1. Supplementary RDEB patient and SCC tumor sample details

Patient	Subtype RDEB*	Sex, Age at the initial diagnosis	Survival after the initial diagnosis (months)	Number of SCCs	Prior treatment	Cause of death	Location, type of SCC	Differentiation classification	**group**	**FFPE**	**RNA**
1	1	M, 43	65	4	None	alive	Hands<br>Primary and recurrence	Well	**Low-risk pSCC**	**√**	
								Well	**Low-risk rSCC**		**√**
								Well	**Low-risk rSCC**	**√**	
2	1	F, 27	109	4	None	alive	Foot, Lower leg<br>Recurrence	Well**	**High-risk rSCC**	**√**	**√**
3	1	F, 25	129	3	None	alive	Lower leg<br>Recurrence	Well	**Low-risk rSCC**	**√**	**√**
4	1	F, 18	168	17	None	gastrointestinal amyloidosis	Foot, Lower leg, Upper leg<br>Recurrence	Well	**Low-risk rSCC**	**√**	
								Well	**Low-risk rSCC**	**√**	
								Well	**Low-risk rSCC**	**√**	
								Well	**Low-risk rSCC**		**√**
5	3	F, 34	253	8	None	alive	Neck<br>Recurrence	Well	**Low-risk rSCC**	**√**	
								Well**	**High-risk rSCC**	**√**	
6	1	M, 28	194	13	None	sepsis	Lower leg<br>Recurrence	Well	**Low-risk rSCC**	**√**	
7	1	M, 31	14	3	None	metastasis	Hands<br>Primary	Poor	**High-risk pSCC**	**√**	**√**
								Moderate	**Low-risk pSCC**	**√**	
8	1	M, 34	175	11	None	alive	Back, Lower leg<br>Rrecurrence	Well	**Low-risk rSCC**	**√**	**√**
								Well	**Low-risk rSCC**		**√**
9	2	M, 33	229	8	None	alive	Low leg<br>Recurrence	Well	**Low-risk rSCC**	**√**	**√**
10	1	F, 27	14	6	None	metastasis	Back<br>Primary, recurrence and metastasis	Poor	**High-risk pSCC**	**√**	
								Moderate	**High-risk rSCC**		**√**
								Well	**High-risk rSCC**	**√**	**√**
								Poor	**High-risk rSCC**	**√**	**√**
11	1	F, 25	16	3	None	extensive SCC	Low leg<br>Primary and recurrence	Well	**High-risk pSCC**	**√**	
								Poor	**High-risk rSCC**	**√**	
12	1	F, 21	60	2	None	alive	Foot, Lower leg<br>Primary and recurrence	Well	**Low-risk pSCC**	**√**	**√**
								Moderate	**Low-risk rSCC**	**√**	
13	1	F, 39	56	2	None	metastasis	Hands<br>Primary and recurrence	Well	**Low-risk pSCC**	**√**	**√**
								Moderate	**High-risk rSCC**		**√**
14	3	F, 33	56	1	None	alive	Neck<br>Primary	Well	**Low-risk pSCC**	**√**	**√**
15	1	F, 27	5	2	None	sepsis	Upper leg<br>Primary	Well	**Low-risk pSCC**	**√**	**√**
16	2	F, 48	137	2	None	alive	Lower leg<br>Recurrence	Well	**Low-risk rSCC**	**√**	**√**
17	1	F, 22	26	3	None	sepsis	Lower leg<br>Primary and recurrence	Poor	**High-risk pSCC**	**√**	**√**
								Moderate	**High-risk rSCC**	**√**	**√**
18	1	M, 20	13	5	None	extensive SCC	Back, Lower leg<br>Primary	Poor	**High-risk pSCC**	**√**	**√**
								Moderate	**High-risk rSCC**	**√**	**√**
								Poor	**High-risk rSCC**	**√**	**√**
19	1	F, 30	28	2	None	alive	Hand<br>Primary	Well	**Low-risk pSCC**		**√**
20	1	F, 31	15	4	None	alive	Upper leg, Foot<br>Recurrence	Well	**Low-risk rSCC**	**√**	
								Well	**Low-risk rSCC**	**√**	

SCCs cutaneous squamous cell carcinomas, FFPE formalin fixed and paraffin embedded, F Female, M Male, LG-pSCC low risk primary SCC, HG-pSCC high-risk primary SCC, LG-rSCC low-risk recurrent SCC, HG-rSCC high-risk recurrent SCC.
* Subtypes of RDEB with 1: severe RDEB, 2: intermediary RDEB, 3: inversed RDEB
** Well differentiated SCC with aggressive criteria (size of SCC or perineural invasion)

Supplementary Table S2. Aggressiveness parameters in high-risk SCC groups

		HR-pSCC (n=5)<br>n (%)	HR-rSCC (*n=10*)<br>n (%)
Local recurrence or metastasis at 3 months		4 (88.0)	6 (60.0)
Massive local expansion – limb amputation recommendation		1 (20.0)	5 (50.0)
Histopathological criteria:	Invasion depth ≥ 6 mm	2 (40.0)	5 (50.0)
	Lympho-vascular space invasion	0 (0.0)	1 (10.0)
	Perineural invasion	0 (0.0)	2 (20.0)
	Poorly differentiated	4 (88.0)	3 (30.0)

HR-pSCC high-risk primary SCC, HR-rSCC high-risk recurrent SCC

Supplementary Table S3. Cox proportional hazards regression of clinical and histopathological variables in association with prognosis of RDEB patients with a primary SCC.

Variable	Low-risk pSCC	High-risk pSCC	Multivariate Cox aHR	p Value
Age at the diagnosis				
1st-3rd quartile (25-34years), n (%)	10/15 (66.6)	3/5 (60.0)	0.99 (0.86-1.15)	0.90
4th quartile (18-48years), n (%)	5/15 (33.3)	2/5 (40.0)		
Gender				
Male, n (%)	4/15 (26.7)	2/5 (40.0)	2.24 (0.34-20.70)	0.42
Female, n (%)	11/15 (73.3)	3/5 (60.0)		
Location				
Lower limb, n (%)	7/15 (40.0)	2/5 (40.0)	1.44 (0.70-3.17)	0.33
Upper limb, n (%)	5/15 (33.3)	1/5 (20.0)		
Back, n (%)	1/15 (6.7)	2/5 (40.0)		
Neck, n (%)	2/15 (13.7)	0/5 (0.0)		
Missing, n (%)	1/15 (6.7)	0/5 (0.0)		
Differentiation				
Good or Moderate, n (%)	15/15 (100.0)	1/5 (20.0)	8.717 (1.34-75.68)	0.026
Poor, n (%)	0/15 (0.0)	4/5 (80.0)		

Supplementary Table S4. Listing of antibodies

Antibody	Source	Reference
KI67	Leica	RTU-Ki67-MM1
CD20	Abcam	ab64088
CD3	Abcam	ab5690
CD4	Dako	M7310
CD8	Abcam	ab4055
Myeloperoxidase	R&D System	MAB3174
Tryptase	Abcam	ab2378
Citrullinated histone H3 (cit R2+R8+R17)	Abcam	ab5103
Rat anti-mouse AlexaFluor 488 IgG	Jackson ImmunoResearch	415-545-166
Donkey anti-rabbit AlexaFluor 594 IgG	Jackson ImmunoResearch	711-585-166
Goat ant-rat AlexaFluor 647 IgG	Jackson ImmunoResearch	112-165-003

Supplementary Table S5. Listing of primers used for RT-quantitative PCR

Gene	Forward Primer	Reverse Primer
CDH1	CGGACGATGATGTGAACACC	TTGCTGTTGTGCTTAACCCC
CSF2	AGCCGACCTGCCTACAGAC	AAGGGGATGACAAGCAGAAA
CSF3	CCACGAATTTGCTGGGGAAT	CTTTCACACACAGGCCTGAC
CXCL8	ACTCCTTGGCAAAACTGCAC	AAACCAAGGCACAGTGGAAC
HMGB1	TAGGGTGGTGTGGAGGAAAC	CTCCCGACAAGTTTGCACAA
IL1β	CTCTGGGATTCTCTTCAGCCA	CCTCATTGCCACTGTAATAAGCC
KRT1	TCAACAAGCGGACAAATGCA	CAATGATGCTGTCCAGGTCG
OCLN	GGGCATTGCTCATCCTGAAG	GAGTAGGCTGGCTGAGAGAG
PADI4	CAGGGGACATTGATCCGTGTG	GGGAGGCGTTGATGCTGAA
PGK	CTGTGGCTTCTGGCATACCT	AATCTGCTTAGCCCGAGTGA

Supplementary Figures

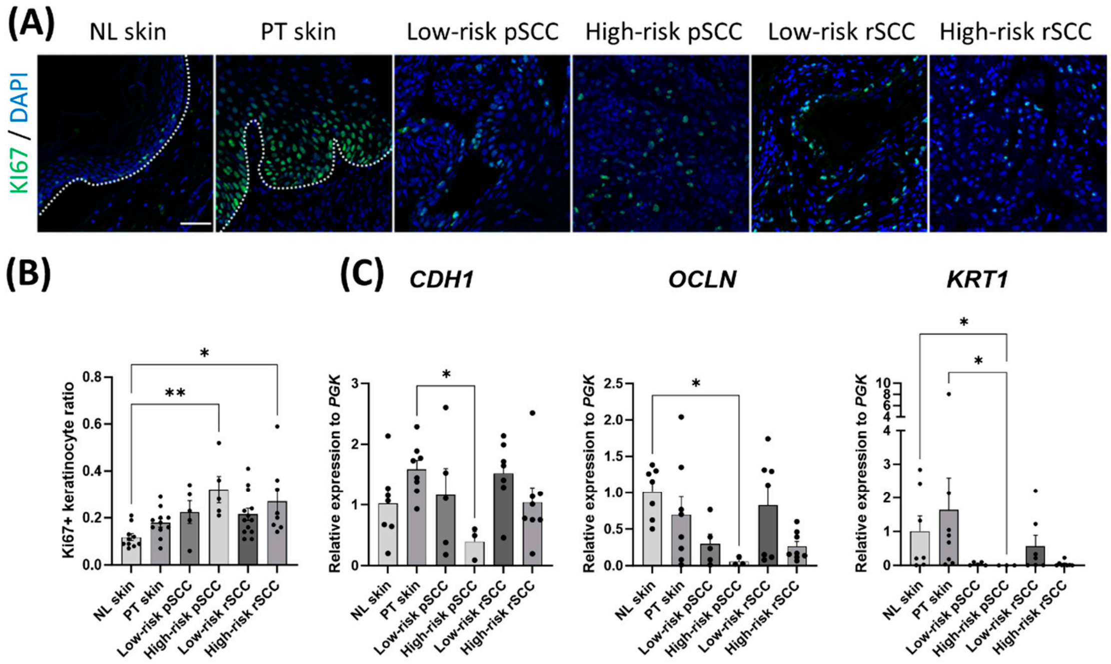

Supplementary Figure S1: Detection of Ki67 and epithelial gene markers in RDEB-SCCs with different severity. (A) Representative immunostaining for Ki67+ cells in in non-lesional skin (NL skin), peri-tumoral skin (PT skin), low-risk primary SCCs (Low-risk pSCC), high-risk primary SCCs (High-risk pSCC), low-risk recurrent SCCs (Low-risk pSCC) and high-risk recurrent SCCs (High-risk rSCC) in RDEB patients, scale bar = 50µm (magnification x400). Scale bar = 50µm. (B) Proportion of Ki67-positive cells compared with the total epithelial cells, n=8 for NL skin, n=11 for PT skin, n=5 for Low-risk pSCC, n=5 for High-risk pSCC, n=13 for Low-risk rSCC, n=8 for High-risk rSCC. (C) Quantitative PCR analysis of *CDH1, OCLN,* and *KRT1* transcripts in NL skin (n=8), PT skin (n=7), Low-risk pSCC (n=6), High-risk pSCC (n=3), Low-risk rSCC (n=7), and High-risk rSCCs (n=8) in RDEB patients in our study. Relative gene expression was normalized to the expression of a housekeeping gene (*PGK)* and expressed as fold-increase compared to NL skin. * p<0.05 Kruskal Willis test with Dunn's or ANOVA with Tukey's multiple comparison post hoc. Data are means ± SEM.

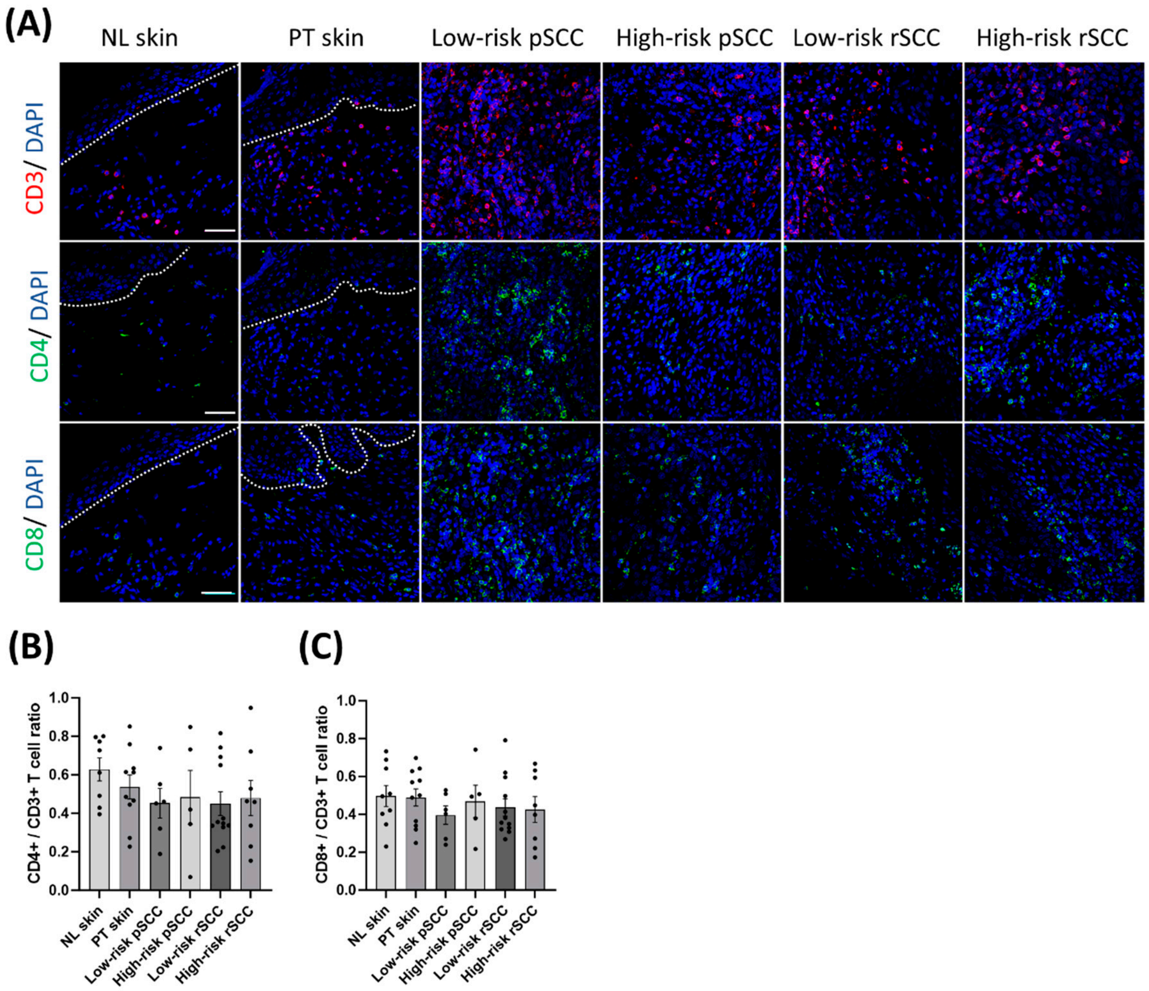

Supplementary Figure S2: Detection of adaptive immune cells in RDEB-SCCs with different severity. (A) Representative immunostaining for infiltrated CD3+ T cells, CD4+ T cells, CD8+ T cells in non-lesional skin (NL skin), peri-tumoral skin (PT skin), low-risk primary SCCs (Low-risk pSCC), high-risk primary SCCs (High-risk pSCC), low-risk recurrent SCCs (Low-risk pSCC) and high-risk recurrent SCCs (High-risk rSCC) in RDEB patients, scale bar = 50µm. Quantification of the number of positive cells per field CD4+ T cell to CD3+ T cell ratio (C), CD8+ T cell to CD3+ T cell ratio (C), n=8 for NL skin, n=12 for PT skin, n=5 for Low-risk pSCC, n=5 for High-risk pSCC, n=13 for Low-risk rSCC, n=8 for High-risk rSCC. * $p<0.05$ Kruskal Wallis test with Dunn's or ANOVA with Tukey's multiple comparison post hoc. Data are means ± SEM.

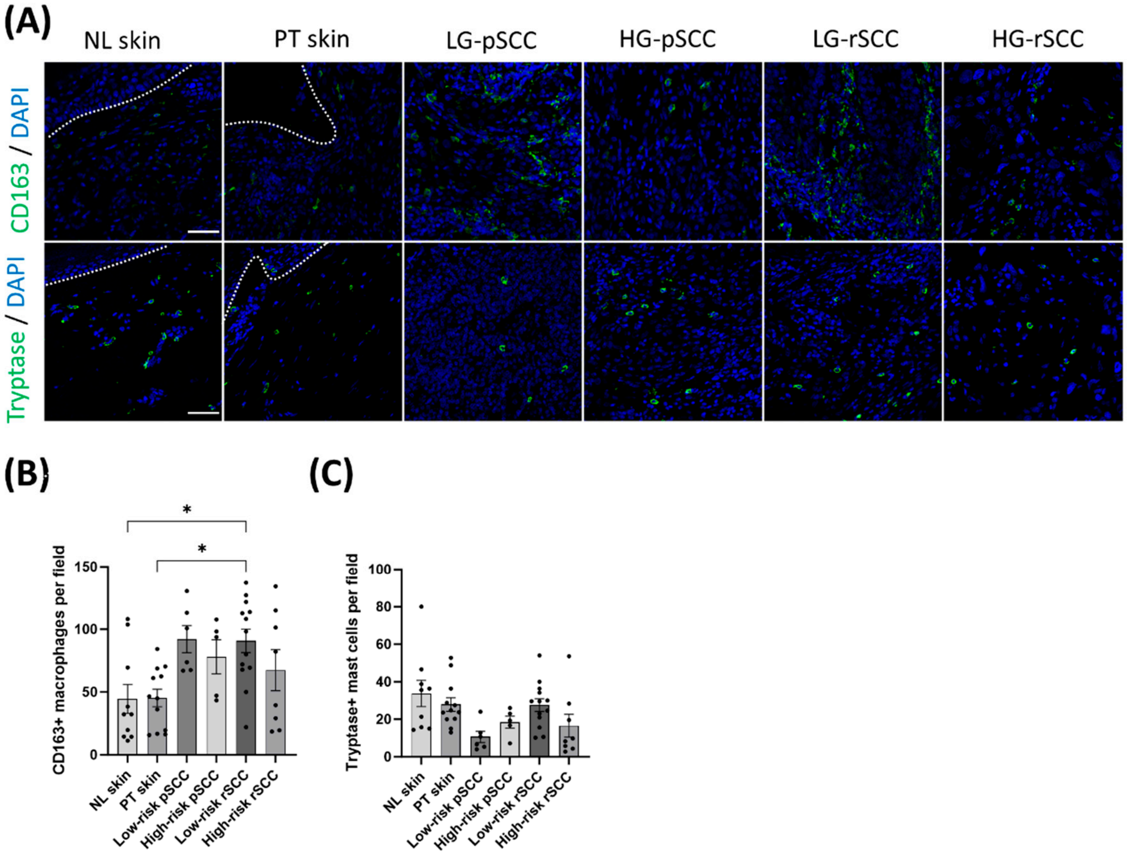

Supplementary Figure S3*: Detection of innate immune cells in RDEB-SCCs with different severity.* (A) Representative immunostaining for infiltrated CD163+ macrophages and tryptase+ mast cells in non-lesional skin (NL skin), peri-tumoral skin (PT skin), low-risk primary SCCs (Low-risk pSCC), high-risk primary SCCs (High-risk pSCC), low-risk recurrent SCCs (Low-risk pSCC) and high-risk recurrent SCCs (High-risk rSCC) in RDEB patients, scale bar = 50µm. Quantification of the number of positive cells per field for CD163+ macrophages (B), tryptase + mast cells (C) in the different conditions. n=8 for NL skin, n=13 for PT skin, n=6 for Low-risk pSCC, n=5 for High-risk pSCC, n=13 for Low-risk rSCC, n=8 for High-risk rSCC. *p<0.05 Kruskal Wallis test with Dunn's or ANOVA with Tukey’s multiple comparison post hoc. Data are means ± SEM.

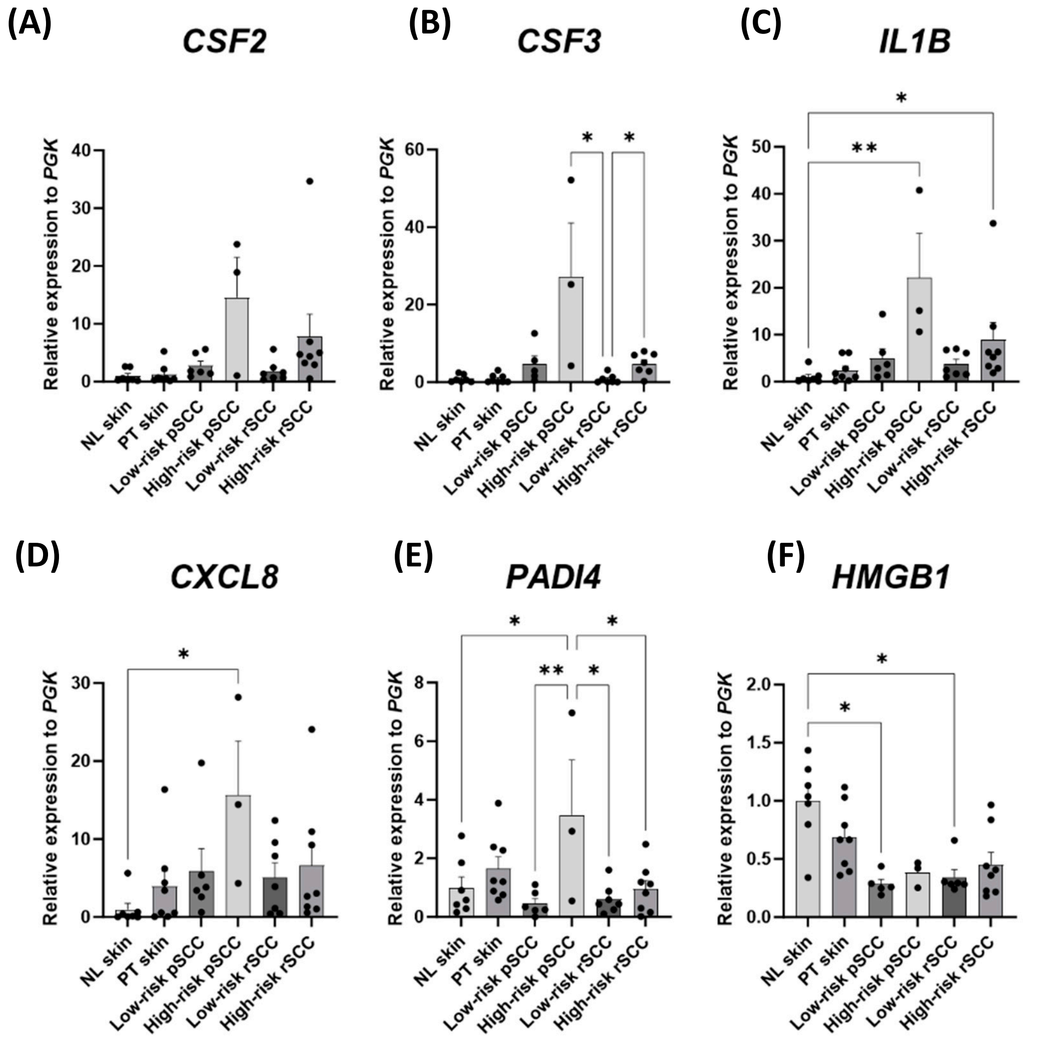

Supplementary Figure S4: Gene expression of inflammatory mediators involved in neutrophil activation in RDEB-SCCs by quantitative RT-PCR. Quantitative PCR analysis of *CSF2* (A), *CSF3* (B), *IL1B* (C), *CXCL8* (D), *PADI4* (E), and *HMGB1* (F) transcripts in non-lesional skin (NL skin) (n=8), peri-tumoral skin (PT skin) (n=7), low-risk primary SCCs (Low-risk pSCC) (n=6), high-risk primary SCCs (High-risk pSCC) (n=3), low-risk recurrent SCCs (Low-risk rSCC) (n=7), and high-risk recurrent SCCs (High-risk rSCC) (n=8) in RDEB patients in our study. Relative gene expression was normalized to the expression of a housekeeping gene (*PGK)* and expressed as fold-increase compared to NL skin. * $p<0.05$ Kruskal Wallis test with Dunn's or ANOVA with Tukey's multiple comparison post hoc. Data are means ± SEM.
